# Association between interpregnancy interval and maternal and neonatal adverse outcomes in women with a cesarean delivery: a population-based study

**DOI:** 10.1186/s12884-023-05600-x

**Published:** 2023-04-25

**Authors:** Hong Dong, Jinghan Chi, Wei Wang, Lei Liu

**Affiliations:** 1 Department of Children’s Medical Center, Sun Yat-Sen Memorial Hospital, Sun Yat-Sen University, Guangzhou, 510120 People’s Republic of China; 2 Senior Department of Pediatrics, The Seventh Medical Center of PLA General Hospital, Beijing, 100700 People’s Republic of China; 3 Department of Radiology, The Seventh Medical Center of PLA General Hospital, Beijing, 100700 People’s Republic of China; 4 Department of Comprehensive Treatment, The Second Medical Center of PLA General Hospital, No. 28 Fuxing Road, Haidian District, Beijing, 100036 People’s Republic of China

**Keywords:** Interpregnancy interval, Cesarean delivery, Maternal outcome, Neonatal events

## Abstract

**Background:**

Interpregnancy interval (IPI) has been linked with several maternal and neonatal adverse events in the general population. However, the association between IPI and maternal and neonatal outcomes in women whose first delivery was by cesarean delivery is unclear. We aimed to investigate the association between IPI after cesarean delivery and the risk of maternal and neonatal adverse events.

**Methods:**

Women (aged ≥ 18 years) whose first delivery was cesarean delivery with 2 consecutive singleton pregnancies from the National Vital Statistics System (NVSS) database between 2017 and 2019 were included in this retrospective cohort study. In this post-hoc analysis, logistic regression analyses were used to examine IPI (≤ 11, 12–17, 18–23 [reference], 24–35, 36–59, and ≥ 60 months) in relation to the risk of repeat cesarean delivery, maternal adverse events (maternal transfusion, ruptured uterus, unplanned hysterectomy, and admission to an intensive care unit), and neonatal adverse events (low birthweight, premature birth, Apgar score at 5 min < 7, and abnormal conditions of the newborn). Stratified analysis based on age (< 35 and ≥ 35 years) and previous preterm birth.

**Results:**

We included 792,094 maternities, 704,244 (88.91%) of which underwent a repeat cesarean delivery, 5,246 (0.66%) women had adverse events, and 144,423 (18.23%) neonates had adverse events. After adjusting for confounders, compared to an IPI of 18–23 months, the IPI of ≤ 11 months [odds ratio (OR) = 1.55, 95% confidence interval (CI): 1.44–1.66], 12–17 months (OR = 1.38, 95%CI: 1.33–1.43), 36–59 months (OR = 1.12, 95%CI: 1.10–1.15), and ≥ 60 months (OR = 1.19, 95%CI: 1.16–1.22) were associated with an increased risk of repeat cesarean delivery. In terms of maternal adverse events, only IPI of ≥ 60 months (OR = 0.85, 95%CI: 0.76–0.95) was observed to be associated with decreased risk of maternal adverse events in women aged < 35 years. In analysis of neonatal adverse events, IPI of ≤ 11 months (OR = 1.14, 95%CI: 1.07–1.21), 12–17 months (OR = 1.07, 95%CI: 1.03–1.10), and ≥ 60 months (OR = 1.05, 95%CI: 1.02–1.08) were related to an increased risk of neonatal adverse events.

**Conclusion:**

Both short and long IPI were associated with an increased risk of repeat cesarean delivery and neonatal adverse events, and women < 35 years may benefit from a longer IPI.

**Supplementary Information:**

The online version contains supplementary material available at 10.1186/s12884-023-05600-x.

## Background

Interpregnancy interval (IPI, intervals between delivery and subsequent conception) is a potentially modifiable risk factor for adverse neonatal and maternal outcomes [1, 2]. Epidemiological evidence suggested that IPI has an impact on maternal and neonatal morbidity [3, 4]. Short IPI (less than 18 months) has been found to be associated with maternal infection and death, preterm birth, small for gestational age and low birth weight infants, neonatal intensive care unit (NICU) admissions, and neonatal death [5–8]. Long IPI (60 months or above) was associated with maternal preeclampsia and gestational diabetes, as well as preterm birth, low birthweight, admission to the NICU, and stillbirth in newborns [2, 5, 9, 10]. Based on these adverse events, the World Health Organization currently recommends that the IPI should be at least 2 years [11].

Previous studies have reported the relationship between IPI and maternal and neonatal outcomes in subsequent pregnancies, but they did not consider the possible impact of the model of the first delivery [5, 9, 12]. The delivery model of the first pregnancy may influence the outcomes of subsequent pregnancies [13]. Clark et al. demonstrated that cesarean delivery was more likely to cause maternal morbidity and mortality than vaginal delivery in low-risk pregnancies [14]. Kjerulff et al. showed that cesarean delivery was also associated with a reduced likelihood of subsequent live birth compared with vaginal delivery [15]. Cesarean delivery is reported to occur in approximately one-third of pregnant women in the United States each year [16]. In addition, women who had a previous cesarean delivery were more likely to have a subsequent cesarean delivery [17], while those who had a previous vaginal birth were more likely to have a subsequent vaginal birth [18]. Several studies have shown that short IPI after cesarean delivery were associated with an increased risk of maternal uterine rupture in subsequent pregnancies [19–21]. However, the effect of IPI after cesarean delivery on other maternal and neonatal outcomes in subsequent pregnancies is poorly understood. And no recommendation exists for the optimal IPI after cesarean delivery.

Therefore, this study aimed to assess the association between IPI after cesarean delivery and the risks of repeat cesarean delivery, maternal and neonatal adverse events in the subsequent pregnancy.

## Methods

### Data source and participants

We conducted a retrospective cohort study and used data from the National Vital Statistics System (NVSS) database between 2017 and 2019. The NVSS database (https://www.cdc.gov/nchs/nvss/about_nvss.htm) is a decentralized, cooperative system completed by the National Center for Health Statistics (NCHS) and 57 registration areas [the 50 States, 2 cities (Washington, DC, and New York City), and 5 territories (Puerto Rico, the Virgin Islands, Guam, American Samoa, and the Commonwealth of the Northern Mariana Islands)] containing vital statistics on birth, deaths, marriages, divorces, and fetal deaths. Medical and health information for maternal and neonatal is included in the medical records. We limited the analytical cohort to the following women: (1) with 2 consecutive singleton pregnancies; (2) whose first delivery was by cesarean delivery; (3) aged ≥ 18 years old; and (4) with assessment of maternal and neonatal outcomes. Women were excluded due to the following criteria: (1) missing information of IPI; (2) gestational age was recorded as < 20 or ≥ 45 weeks; (3) with multifetal pregnancies or stillbirths; and (4) missing information of key covariates such as weight gain, pre-pregnancy body mass index (BMI), smoking status, etc. Because the medical records in the NVSS database are publicly available and the data are de-identified, this post-hoc analysis study was exempt from institutional review board approval.

### Study outcomes

The primary outcome of this study was the effect of IPI on the risk of repeat cesarean delivery in pregnant women. The secondary outcomes were maternal adverse events and neonatal adverse events. IPI was defined as the time elapsed between a women’s first live birth and her next pregnancy, which was estimated by the delivery data of the second neonatal minus its gestational age at birth. IPI was categorized into ≤ 11 months, 12–17 months, 18–23 months, 24–35 months, 36–59 months, and ≥ 60 months, with 18–23 months as the reference category [22]. Maternal adverse events included maternal transfusion, ruptured uterus, unplanned hysterectomy, and admission to an intensive care unit (ICU), and the occurrence of one of these events was defined as maternal adverse events. Neonatal adverse events included low birthweight (birth weight less than 2,500 g), premature birth (delivery less than 37 completed weeks of gestation), Apgar score at 5 min < 7, and abnormal conditions of the newborn (assisted ventilation required immediately following delivery, assisted ventilation required for more than six hours, NICU admission, newborn given surfactant replacement therapy, antibiotics received by the newborn for suspected neonatal sepsis, seizure or serious neurologic dysfunction), and the occurrence of one of these events was defined as neonatal adverse events.

### Variables

Data on maternal second singleton pregnancies were collected. Maternal characteristics included age, race (White, Black, Asian, and others), education level (less than high school, high school, more than high school, and missing), marital status (married, unmarried, and missing), weight gain, smoking before pregnancy (yes and no), smoking during pregnancy (yes and no), prenatal care (yes and no), pre-pregnancy BMI, pre-pregnancy diabetes (yes and no), gestational diabetes (yes and no), pre-pregnancy hypertension (yes and no), gestation hypertension (yes and no), eclampsia (yes and no), assisted reproductive treatment (yes and no), gestational age, clinical chorioamnionitis or maternal fever during labor (yes and no), previous preterm birth (yes and no), method of delivery (vaginal delivery and cesarean delivery), and maternal adverse events (maternal transfusion, ruptured uterus, unplanned hysterectomy, and admission to ICU). Neonatal characteristics collected neonatal adverse events (low birth weight, premature birth, Apgar score at 5 min < 7, and abnormal conditions of the newborn).

### Statistical analysis

Data were expressed as mean and standard deviation (SD), median and quartile [M (Q1, Q3)] or number and percentage [n (%)]. Student’s t test, analysis of variance, and Kruskal–Wallis test were used for the comparison of quantitative data between groups. Chi-square test or rank sum test was used for the comparison of enumeration data. Univariate logistic regression analysis was utilized to screen for confounders associated with cesarean delivery, maternal and neonatal adverse events, where statistically significant variables were included as confounders for adjustment in multivariate logistic regression analysis. The covariates related to cesarean delivery, maternal adverse events, and neonatal adverse events were similar in the univariate logistic regression analysis and thus the same confounders were adjusted for in the multivariate logistic regression analysis (Supplement Tables 1–3). Multivariate logistic regression analysis was used to analyze the association between IPI and the risks of cesarean delivery, maternal and neonatal adverse events by adjusting for age, race, education level, marital status, weight gain, smoking before pregnancy, smoking during pregnancy, prenatal care, pre-pregnancy BMI, pre-pregnancy diabetes, gestational diabetes, pre-pregnancy hypertension, gestation hypertension, eclampsia, assisted reproductive treatment, gestational age, clinical chorioamnionitis or maternal fever during labor, and previous preterm birth. Odds ratio (OR) and 95% confidence interval (CI) were used to assess effect values. Stratified analyses based on maternal age (< 35 and ≥ 35 years) and previous preterm birth were used to further analyze the relationship between IPI and the risk of cesarean delivery, maternal and neonatal adverse events. Analyses were performed with the SAS 9.3 software (SAS Institute Inc., Cary, NC, USA). Statistical tests were two-sided, and *P* < 0.05 indicates statistical significance.

**Table 1:** Characteristics of the study population by interpregnancy interval (IPI)

		**Interpregnancy interval**							
**Variables**	Total (n = 792,094)	≤ 11 month (n = 12,176)	12–17 month (n = 56,716)	18–23 month (n = 97,840)	24–35 month (n = 186,588)	36–59 month (n = 208,005)	≥ 60 month (n = 230,769)	**Statistics**	***P***
Age, year, mean ± SD	30.42 ± 5.36	26.53 ± 5.70	28.14 ± 5.76	29.69 ± 5.52	30.36 ± 5.33	30.32 ± 5.29	31.64 ± 4.90	F = 6357.89	< 0.001
Race, n (%)									
White	578,146 (72.99)	8166 (67.07)	41,196 (72.64)	76,140 (77.82)	145,666 (78.07)	152,414 (73.27)	154,564 (66.98)	χ^2^ = 12,146.74	< 0.001
Black	116,083 (14.66)	2823 (23.18)	9904 (17.46)	11,748 (12.01)	19,647 (10.53)	27,413 (13.18)	44,548 (19.30)		
Other	24,995 (3.16)	578 (4.75)	2355 (4.15)	3031 (3.10)	5362 (2.87)	6262 (3.01)	7407 (3.21)		
Asian	72,870 (9.20)	609 (5.00)	3261 (5.75)	6921 (7.07)	15,913 (8.53)	21,916 (10.54)	24,250 (10.51)		
Education level, n (%)									
Less than high school	15,163 (1.91)	239 (1.96)	860 (1.52)	1263 (1.29)	2249 (1.21)	3624 (1.74)	6928 (3.00)	χ^2^ = 15,553.44	< 0.001
High school	233,851 (29.52)	5902 (48.47)	20,446 (36.05)	24,433 (24.97)	42,785 (22.93)	58,589 (28.17)	81,696 (35.40)		
More than high school	534,388 (67.47)	5931 (48.71)	34,881 (61.50)	71,244 (72.82)	139,611 (74.82)	143,536 (69.01)	139,185 (60.31)		
Missing	8692 (1.10)	104 (0.85)	529 (0.93)	900 (0.92)	1943 (1.04)	2256 (1.08)	2960 (1.28)		
Marital status, n (%)									
Married	460,078 (58.08)	5215 (42.83)	30,791 (54.29)	63,787 (65.20)	125,514 (67.27)	125,909 (60.53)	108,862 (47.17)	χ^2^ = 26,705.16	< 0.001
Unmarried	224,320 (28.32)	5787 (47.53)	19,419 (34.24)	22,027 (22.51)	36,328 (19.47)	53,066 (25.51)	87,693 (38.00)		
Missing	107,696 (13.60)	1174 (9.64)	6506 (11.47)	12,026 (12.29)	24,746 (13.26)	29,030 (13.96)	34,214 (14.83)		
Weight gain, pounds, M (Q_1_, Q_3_)	28.00 (19.00,37.00)	26.00 (17.00,35.00)	27.00 (18.00,35.00)	29.00 (20.00,37.00)	29.00 (20.00,37.00)	28.00 (19.00,37.00)	28.00 (18.00,38.00)	χ^2^ = 1245.86	< 0.001
Smoking before pregnancy, n (%)									
No	733,610 (92.62)	10,927 (89.74)	52,364 (92.33)	92,779 (94.83)	177,324 (95.04)	193,877 (93.21)	206,339 (89.41)	χ^2^ = 6017.56	< 0.001
Yes	58,484 (7.38)	1249 (10.26)	4352 (7.67)	5061 (5.17)	9264 (4.96)	14,128 (6.79)	24,430 (10.59)		
Smoking during pregnancy, n (%)									
No	747,537 (94.37)	11,120 (91.33)	53,186 (93.78)	93,946 (96.02)	179,767 (96.34)	197,581 (94.99)	211,937 (91.84)	χ^2^ = 5055.38	< 0.001
Yes	44,557 (5.63)	1056 (8.67)	3530 (6.22)	3894 (3.98)	6821 (3.66)	10,424 (5.01)	18,832 (8.16)		
Prenatal care, n (%)									
No	6562 (0.83)	230 (1.89)	695 (1.23)	805 (0.82)	1240 (0.66)	1485 (0.71)	2107 (0.91)	χ^2^ = 389.80	< 0.001
Yes	785,532 (99.17)	11,946 (98.11)	56,021 (98.77)	97,035 (99.18)	185,348 (99.34)	206,520 (99.29)	228,662 (99.09)		
Pre-pregnancy BMI, kg/m^2^, mean ± SD	28.66 ± 7.37	30.20 ± 7.88	29.06 ± 7.48	27.97 ± 7.11	27.80 ± 7.03	28.59 ± 7.32	29.54 ± 7.60	F = 1487.17	< 0.001
Pre-pregnancy diabetes, n (%)									
No	779,451 (98.40)	11,951 (98.15)	55,896 (98.55)	96,627 (98.76)	184,228 (98.74)	204,914 (98.51)	225,835 (97.86)	χ^2^ = 670.14	< 0.001
Yes	12,643 (1.60)	225 (1.85)	820 (1.45)	1213 (1.24)	2360 (1.26)	3091 (1.49)	4934 (2.14)		
Gestational diabetes, n (%)									
No	725,733 (91.62)	11,243 (92.34)	52,746 (93.00)	90,994 (93.00)	173,293 (92.87)	190,448 (91.56)	207,009 (89.70)	χ^2^ = 1879.98	< 0.001
Yes	66,361 (8.38)	933 (7.66)	3970 (7.00)	6846 (7.00)	13,295 (7.13)	17,557 (8.44)	23,760 (10.30)		
Pre-pregnancy hypertension, n (%)									
No	769,955 (97.21)	11,751 (96.51)	55,199 (97.33)	95,701 (97.81)	182,528 (97.82)	202,763 (97.48)	222,013 (96.21)	χ^2^ = 1327.37	< 0.001
Yes	22,139 (2.79)	425 (3.49)	1517 (2.67)	2139 (2.19)	4060 (2.18)	5242 (2.52)	8756 (3.79)		
Gestation hypertension, n (%)									
No	741,155 (93.57)	11,234 (92.26)	53,157 (93.72)	92,370 (94.41)	176,039 (94.35)	194,814 (93.66)	213,541 (92.53)	χ^2^ = 752.12	< 0.001
Yes	50,939 (6.43)	942 (7.74)	3559 (6.28)	5470 (5.59)	10,549 (5.65)	13,191 (6.34)	17,228 (7.47)		
Eclampsia, n (%)									
No	790,160 (99.76)	12,139 (99.70)	56,578 (99.76)	97,636 (99.79)	186,257 (99.82)	207,509 (99.76)	230,041 (99.68)	χ^2^ = 89.49	< 0.001
Yes	1934 (0.24)	37 (0.30)	138 (0.24)	204 (0.21)	331 (0.18)	496 (0.24)	728 (0.32)		
Assisted reproductive treatment, n (%)									
No	782,824 (98.83)	12,145 (99.75)	56,377 (99.40)	96,504 (98.63)	183,671 (98.44)	205,346 (98.72)	228,781 (99.14)	χ^2^ = 741.76	< 0.001
Yes	9270 (1.17)	31 (0.25)	339 (0.60)	1336 (1.37)	2917 (1.56)	2659 (1.28)	1988 (0.86)		
Gestational age, week, mean ± SD	38.52 ± 1.96	38.02 ± 2.55	38.53 ± 2.08	38.62 ± 1.88	38.65 ± 1.80	38.55 ± 1.89	38.36 ± 2.08	F = 670.30	< 0.001
Clinical chorioamnionitis or maternal fever during labor, n (%)									
No	786,200 (99.26)	12,125 (99.58)	56,379 (99.41)	97,105 (99.25)	185,227 (99.27)	206,399 (99.23)	228,965 (99.22)	χ^2^ = 41.94	< 0.001
Yes	5894 (0.74)	51 (0.42)	337 (0.59)	735 (0.75)	1361 (0.73)	1606 (0.77)	1804 (0.78)		
Previous preterm birth, n (%)									
No	754,627 (95.27)	11,241 (92.32)	53,573 (94.46)	93,460 (95.52)	178,306 (95.56)	198,460 (95.41)	219,587 (95.15)	χ^2^ = 383.01	< 0.001
Yes	37,467 (4.73)	935 (7.68)	3143 (5.54)	4380 (4.48)	8282 (4.44)	9545 (4.59)	11,182 (4.85)		
Method of delivery, n (%)									
Vaginal delivery	87,850 (11.09)	1010 (8.30)	5515 (9.72)	12,788 (13.07)	24,303 (13.02)	23,019 (11.07)	21,215 (9.19)	χ^2^ = 2143.50	< 0.001
Cesarean delivery	704,244 (88.91)	11,166 (91.70)	51,201 (90.28)	85,052 (86.93)	162,285 (86.98)	184,986 (88.93)	209,554 (90.81)		
Maternal adverse events, n (%)									
No	786,848 (99.34)	12,071 (99.14)	56,301 (99.27)	97,182 (99.33)	185,404 (99.37)	206,686 (99.37)	229,204 (99.32)	χ^2^ = 17.29	0.004
Yes	5246 (0.66)	105 (0.86)	415 (0.73)	658 (0.67)	1184 (0.63)	1319 (0.63)	1565 (0.68)		
Maternal transfusion, n (%)									
No	788,712 (99.57)	12,112 (99.47)	56,459 (99.55)	97,387 (99.54)	185,801 (99.58)	207,156 (99.59)	229,797 (99.58)	χ^2^ = 8.72	0.121
Yes	3382 (0.43)	64 (0.53)	257 (0.45)	453 (0.46)	787 (0.42)	849 (0.41)	972 (0.42)		
Ruptured uterus, n (%)									
No	791,312 (99.90)	12,156 (99.84)	56,630 (99.85)	97,719 (99.88)	186,391 (99.89)	207,814 (99.91)	230,602 (99.93)	χ^2^ = 45.72	< 0.001
Yes	782 (0.10)	20 (0.16)	86 (0.15)	121 (0.12)	197 (0.11)	191 (0.09)	167 (0.07)		
Unplanned hysterectomy, n (%)									
No	791,503 (99.93)	12,169 (99.94)	56,677 (99.93)	97,769 (99.93)	186,457 (99.93)	207,846 (99.92)	230,585 (99.92)	χ^2^ = 2.18	0.823
Yes	591 (0.07)	7 (0.06)	39 (0.07)	71 (0.07)	131 (0.07)	159 (0.08)	184 (0.08)		
Admission to ICU, n (%)									
No	790,665 (99.82)	12,150 (99.79)	56,619 (99.83)	97,698 (99.85)	186,309 (99.85)	207,636 (99.82)	230,253 (99.78)	χ^2^ = 41.67	< 0.001
Yes	1429 (0.18)	26 (0.21)	97 (0.17)	142 (0.15)	279 (0.15)	369 (0.18)	516 (0.22)		
Neonatal adverse events, n(%)									
No	647,671 (81.77)	8891 (73.02)	45,712 (80.60)	81,515 (83.31)	156,834 (84.05)	171,927 (82.66)	182,792 (79.21)	χ^2^ = 2610.49	< 0.001
Yes	144,423 (18.23)	3285 (26.98)	11,004 (19.40)	16,325 (16.69)	29,754 (15.95)	36,078 (17.34)	47,977 (20.79)		
Low birth weight, n (%)									
No	749,746 (94.65)	11,026 (90.56)	53,728 (94.73)	93,789 (95.86)	179,043 (95.96)	197,677 (95.03)	214,483 (92.94)	χ^2^ = 2706.29	< 0.001
Yes	42,348 (5.35)	1150 (9.44)	2988 (5.27)	4051 (4.14)	7545 (4.04)	10,328 (4.97)	16,286 (7.06)		
Premature birth, n (%)									
No	714,221 (90.17)	10,049 (82.53)	50,613 (89.24)	89,233 (91.20)	171,435 (91.88)	188,987 (90.86)	203,904 (88.36)	χ^2^ = 2554.33	< 0.001
Yes	77,873 (9.83)	2127 (17.47)	6103 (10.76)	8607 (8.80)	15,153 (8.12)	19,018 (9.14)	26,865 (11.64)		
Apgar score at 5 min < 7, n (%)									
No	781,028 (98.60)	11,905 (97.77)	55,861 (98.49)	96,595 (98.73)	184,295 (98.77)	205,258 (98.68)	227,114 (98.42)	χ^2^ = 182.29	< 0.001
Yes	11,066 (1.40)	271 (2.23)	855 (1.51)	1245 (1.27)	2293 (1.23)	2747 (1.32)	3655 (1.58)		
Abnormal conditions of the newborn, n (%)									
No	707,257 (89.29)	10,334 (84.87)	50,395 (88.85)	88,285 (90.23)	168,667 (90.40)	186,685 (89.75)	202,891 (87.92)	χ^2^ = 1088.64	< 0.001
Yes	84,837 (10.71)	1842 (15.13)	6321 (11.15)	9555 (9.77)	17,921 (9.60)	21,320 (10.25)	27,878 (12.08)		

## Results

### Characteristics of participants

**Fig. 1:**
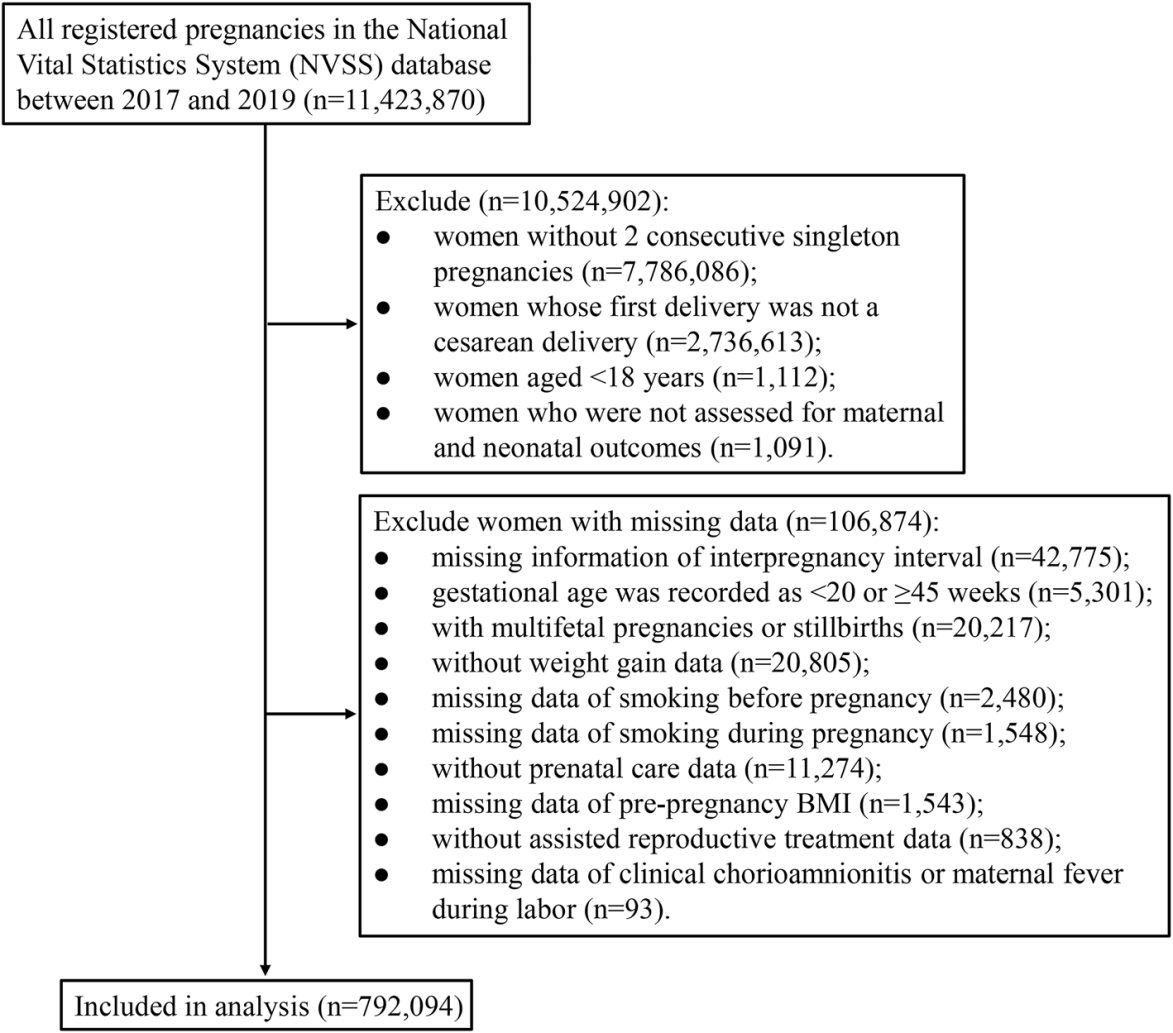
Flowchart showing the selection of the study population

**Fig. 2:**
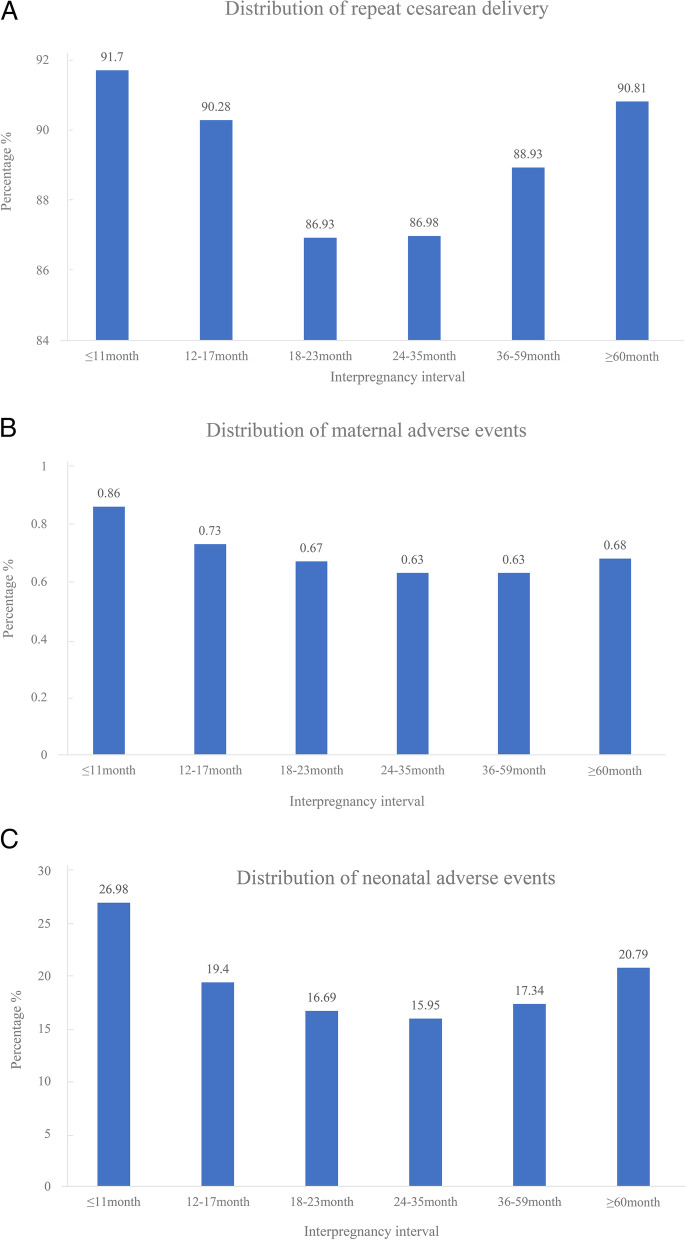
The proportions of repeat cesarean delivery, maternal adverse events, and neonatal adverse events by interpregnancy interval (IPI). **A** distribution of repeat cesarean delivery; **B** distribution of maternal adverse events; **C** distribution of neonatal adverse events

There were 11,423,870 maternities in the NVSS database during our study period. After excluding 7,786,080 maternities who did not have 2 consecutive singleton pregnancies, 2,736,613 maternities whose first delivery was not a cesarean delivery, 1,112 maternities younger than 18 years of age, 1,091 maternities who were not assessed for maternal and neonatal outcomes, and 106,874 maternities who were missing key covariates, a total of 792,094 maternities were included in this study (Fig. 1). Of these 792,094 women, the mean age was 30.42 ± 5.36 years, the mean pre-pregnancy BMI was 28.66 ± 7.37 kg/m^2^, and 578,146 (72.99%) were white. Approximately 1.5% (12,176) of deliveries occurred after an IPI of ≤ 11 months, 7.2% (56,716) after an IPI of 12–17 months, 12.4% (97,840) after an IPI of 18–23 months, 23.5% (186,588) after an IPI of 24–35 months, 26.3% (208,005) after an IPI of 36–59 months, and 29.1% (230,769) after an IPI of ≥ 60 months. There were 37,467 (4.73%) women who had a previous preterm birth, 704,244 (88.91%) women who underwent a repeat cesarean delivery, 5,246 (0.66%) women who had adverse events, and 144,423 (18.23%) neonates who had adverse events (Table 1). The proportions of repeat cesarean delivery, maternal adverse events, and neonatal adverse events by IPI were shown in Fig. 2.

### Effect of IPI on the risk of repeat cesarean delivery

Table 2 shows the association between IPI and the risk of repeat cesarean delivery. After adjusting for confounders, compared to an IPI of 18–23 months, the IPI of ≤ 11 months (OR = 1.55, 95%CI: 1.44–1.66), 12–17 months (OR = 1.38, 95%CI: 1.33–1.43), 36–59 months (OR = 1.12, 95%CI: 1.10–1.15), and ≥ 60 months (OR = 1.19, 95%CI: 1.16–1.22) were associated with an increased risk of repeat cesarean delivery, whereas an IPI of 24–35 months may not be related to a risk of repeat cesarean delivery (*P* = 0.159). Among women of different ages, the IPI of ≤ 11 months, 12–17 months, 36–59 months, and ≥ 60 months were still associated with an increased risk of repeat cesarean delivery in maternities aged < 35 years (all *P* < 0.001), while an IPI of 24–35 months (OR = 0.92, 95%CI: 0.86–0.98) was related to a decreased risk of repeat cesarean delivery in maternities aged ≥ 35 years. Among women with and without previous preterm births, the IPI of ≤ 11 months, 12–17 months, 36–59 months, and ≥ 60 months were associated with an increased risk of repeat cesarean delivery (all *P* < 0.05).

**Table 2:** Association between interpregnancy interval (IPI) and the risk of repeat cesarean delivery

Cesarean delivery	Interpregnancy interval										
	≤ 11 month		12–17 month		18–23 month	24–35 month		36–59 month		≥ 60 month	
	OR (95% CI)	*P*	OR (95% CI)	*P*	OR (95% CI)	OR (95% CI)	*P*	OR (95% CI)	*P*	OR (95% CI)	*P*
Total population	1.55 (1.44–1.66)	< 0.001	1.38 (1.33–1.43)	< 0.001	1.00 [Ref]	0.98 (0.96–1.01)	0.159	1.12 (1.10–1.15)	< 0.001	1.19 (1.16–1.22)	< 0.001
Age group											
< 35	1.47 (1.36–1.58)	< 0.001	1.34 (1.29–1.40)	< 0.001	1.00 [Ref]	1.01 (0.98–1.04)	0.431	1.17 (1.14–1.21)	< 0.001	1.28 (1.24–1.31)	< 0.001
≥ 35	1.77 (1.34–2.33)	< 0.001	1.37 (1.24–1.53)	< 0.001	1.00 [Ref]	0.92 (0.86–0.98)	0.008	1.02 (0.96–1.09)	0.460	1.23 (1.15–1.31)	< 0.001
Previous preterm births group											
No	1.56 (1.44–1.68)	< 0.001	1.38 (1.33–1.44)	< 0.001	1.00 [Ref]	0.98 (0.96–1.01)	0.162	1.12 (1.09–1.15)	< 0.001	1.20 (1.16–1.23)	< 0.001
Yes	1.52 (1.20–1.92)	< 0.001	1.29 (1.12–1.48)	< 0.001	1.00 [Ref]	0.98 (0.88–1.09)	0.754	1.14 (1.03–1.27)	0.014	1.12 (1.01–1.25)	0.035

*OR* odds ratio, *CI* confidence interval, *Ref* reference; OR shows the results of multivariate logistic regression analysis after adjusting for age (unadjusted in age group analysis), race, education level, marital status, weight gain, smoking before pregnancy, smoking during pregnancy, prenatal care, pre-pregnancy BMI, pre-pregnancy diabetes, gestational diabetes, pre-pregnancy hypertension, gestation hypertension, eclampsia, assisted reproductive treatment, gestational age, clinical chorioamnionitis or maternal fever during labor, and previous preterm birth (unadjusted in previous preterm births group analysis)

The association between IPI and the risk of repeat cesarean delivery was further analyzed in women who underwent a trial of labor after cesarean (TOLAC). A total of 133,970 women underwent TOLAC, of which 46,042 (34.37%) women underwent repeat cesarean delivery (Table 3). After adjusting for confounders, compared to an IPI of 18–23 months, the IPI of ≤ 11 months (OR = 1.21, 95%CI: 1.08–1.35), 12–17 months (OR = 1.15, 95%CI: 1.08–1.21), 36–59 months (OR = 1.05, 95%CI: 1.01–1.10), and ≥ 60 months (OR = 1.16, 95%CI: 1.11–1.21) were still related to an increased risk of repeat cesarean delivery in women who underwent TOLAC, while an IPI of 24–35 months may not be related to a risk of repeat cesarean delivery (*P* = 0.483).

**Table 3:** Association between interpregnancy interval (IPI) and risk of repeat cesarean delivery in women who underwent a trial of labor after cesarean (TOLAC)

Variables	Women with TOLAC (n)	Repeat cesarean delivery (%)	Univariate analysis		Multivariate analysis	
			OR (95%CI)	*P*	OR (95%CI)	*P*
	133,970	46,042 (34.37%)				
Interpregnancy interval						
18–23 month	18,564	5765 (31.05%)	Ref		Ref	
≤ 11 month	1593	582 (36.53%)	1.28 (1.15–1.42)	< 0.001	1.21 (1.08–1.35)	0.001
12–17 month	8506	2987 (35.12%)	1.20 (1.14–1.27)	< 0.001	1.15 (1.08–1.21)	< 0.001
24–35 month	35,344	11,018 (31.17%)	1.01 (0.97–1.04)	0.777	0.99 (0.95–1.03)	0.483
36–59 month	34,923	11,881 (34.02%)	1.14 (1.10–1.19)	< 0.001	1.05 (1.01–1.10)	0.011
≥ 60 month	35,040	13,809 (39.41%)	1.44 (1.39–1.50)	< 0.001	1.16 (1.11–1.21)	< 0.001

*OR* odds ratio, *CI* confidence interval, *Ref* reference

Multivariate logistic regression analysis adjusted for age, race, education level, marital status, weight gain, smoking before pregnancy, smoking during pregnancy, prenatal care, pre-pregnancy BMI, pre-pregnancy diabetes, gestational diabetes, pre-pregnancy hypertension, gestation hypertension, eclampsia, assisted reproductive treatment, gestational age, clinical chorioamnionitis or maternal fever during labor, and previous preterm birth

### Association between IPI and maternal adverse events

The impact of IPI on maternal adverse events was demonstrated in Table 4. After adjusting for confounders, IPI of ≤ 11 months, 12–17 months, 24–35 months, 36–59 months, and ≥ 60 months may not be associated with the risk of maternal adverse events compared with IPI of 18–23 months (all *P* > 0.05). Stratified analyses based on age and previous preterm births showed that IPI of ≥ 60 months (OR = 0.85, 95%CI: 0.76–0.95) was observed to be associated with decreased risk of maternal adverse events only in women aged < 35 years.

**Table 4:** Effect of interpregnancy interval (IPI) on maternal adverse events

Outcome	Interpregnancy interval										
	≤ 11 month		12 ~ 17 month		18 ~ 23 month	24 ~ 35 month		36 ~ 59 month		≥ 60 month	
	OR (95% CI)	*P*	OR (95% CI)	*P*	OR (95% CI)	OR (95% CI)	*P*	OR (95% CI)	*P*	OR (95% CI)	*P*
**Maternal adverse events**											
Total population	1.01 (0.81–1.25)	0.919	1.01 (0.89–1.15)	0.827	1.00 [Ref]	0.95 (0.86–1.05)	0.347	0.92 (0.84–1.02)	0.102	0.91 (0.82–0.99)	0.052
Age group											
< 35	1.07 (0.86–1.34)	0.544	1.03 (0.90–1.18)	0.680	1.00 [Ref]	0.91 (0.82–1.02)	0.115	0.91 (0.82–1.02)	0.108	0.85 (0.76–0.95)	0.004
≥ 35	0.52 (0.21–1.29)	0.161	0.90 (0.64–1.27)	0.549	1.00 [Ref]	1.11 (0.89–1.39)	0.361	0.95 (0.76–1.18)	0.620	1.03 (0.83–1.28)	0.771
Previous preterm births group											
No	1.05 (0.83–1.32)	0.694	0.99 (0.87–1.14)	0.955	1.00 [Ref]	0.97 (0.87–1.08)	0.577	0.94 (0.84–1.04)	0.219	0.92 (0.83–1.03)	0.137
Yes	0.77 (0.43–1.37)	0.371	1.11 (0.77–1.60)	0.574	1.00 [Ref]	0.80 (0.59–1.10)	0.179	0.78 (0.57–1.06)	0.116	0.76 (0.56–1.03)	0.080
**Maternal transfusion**											
Total population	0.90 (0.68–1.18)	0.435	0.91 (0.78–1.07)	0.265	1.00 [Ref]	0.92 (0.82–1.04)	0.182	0.88 (0.78–0.99)	0.033	0.86 (0.76–0.97)	0.015
Age group											
< 35	0.99 (0.75–1.30)	0.923	0.97 (0.81–1.14)	0.681	1.00 [Ref]	0.90 (0.79–1.03)	0.123	0.90 (0.79–1.02)	0.110	0.82 (0.72–0.94)	0.004
≥ 35	0.31 (0.08–1.25)	0.100	0.66 (0.42–1.03)	0.069	1.00 [Ref]	0.98 (0.75–1.27)	0.868	0.77 (0.59–1.01)	0.057	0.87 (0.67–1.12)	0.285
Previous preterm births group											
No	0.90 (0.67–1.21)	0.482	0.90 (0.76–1.06)	0.211	1.00 [Ref]	0.94 (0.83–1.07)	0.332	0.88 (0.77–0.99)	0.036	0.87 (0.77–0.99)	0.032
Yes	0.86 (0.41–1.80)	0.688	1.03 (0.64–1.68)	0.894	1.00 [Ref]	0.73 (0.48–1.11)	0.143	0.89 (0.60–1.32)	0.563	0.75 (0.50–1.12)	0.154
**Ruptured uterus**											
Total population	1.47 (0.91–2.38)	0.116	1.27 (0.95–1.70)	0.107	1.00 [Ref]	0.86 (0.68–1.09)	0.212	0.79 (0.63–1.01)	0.059	0.64 (0.50–0.83)	< 0.001
Age group											
< 35	1.52 (0.92–2.50)	0.099	1.26 (0.93–1.72)	0.140	1.00 [Ref]	0.79 (0.61–1.03)	0.083	0.77 (0.59–0.99)	0.046	0.59 (0.45–0.78)	< 0.001
≥ 35	0.95 (0.13–7.21)	0.961	1.28 (0.57–2.92)	0.549	1.00 [Ref]	1.18 (0.66–2.11)	0.573	0.91 (0.50–1.64)	0.749	0.84 (0.46–1.51)	0.557
Previous preterm births group											
No	1.54 (0.91–2.58)	0.105	1.27 (0.93–1.73)	0.127	1.00 [Ref]	0.84 (0.65–1.08)	0.168	0.80 (0.62–1.03)	0.087	0.65 (0.50–0.86)	0.002
Yes	1.09 (0.30–3.99)	0.900	1.24 (0.52–2.94)	0.626	1.00 [Ref]	1.05 (0.51–2.17)	0.898	0.73 (0.34–1.56)	0.415	0.58 (0.26–1.29)	0.181
**Unplanned hysterectomy**											
Total population	0.79 (0.36–1.74)	0.562	0.89 (0.58–1.37)	0.604	1.00 [Ref]	0.96 (0.70–1.30)	0.769	1.03 (0.76–1.38)	0.861	0.92 (0.68–1.24)	0.586
Age group											
< 35	0.71 (0.30–1.66)	0.424	0.77 (0.47–1.27)	0.308	1.00 [Ref]	0.91 (0.63–1.30)	0.590	1.02 (0.72–1.45)	0.914	0.99 (0.70–1.42)	0.977
≥ 35	0.83 (0.11–6.26)	0.853	1.17 (0.52–2.63)	0.706	1.00 [Ref]	1.16 (0.66–2.04)	0.617	1.17 (0.67–2.04)	0.578	1.18 (0.69–2.04)	0.543
Previous preterm births group											
No	0.66 (0.26–1.64)	0.369	0.92 (0.59–1.42)	0.693	1.00 [Ref]	0.92 (0.67–1.27)	0.624	1.03 (0.76–1.39)	0.863	0.90 (0.66–1.22)	0.485
Yes	1.66 (0.29–9.51)	0.568	0.68 (0.12–3.77)	0.662	1.00 [Ref]	1.41 (0.45–4.44)	0.560	1.01 (0.31–3.32)	0.982	1.24 (0.40–3.88)	0.714
**Admission to ICU**											
Total population	0.91 (0.57–1.45)	0.687	1.08 (0.82–1.42)	0.593	1.00 [Ref]	1.02 (0.82–1.27)	0.828	1.13 (0.92–1.39)	0.257	1.16 (0.94–1.42)	0.157
Age group											
< 35	0.89 (0.54–1.46)	0.640	1.07 (0.79–1.45)	0.645	1.00 [Ref]	0.98 (0.77–1.25)	0.870	1.11 (0.88–1.40)	0.381	1.10 (0.87–1.39)	0.426
≥ 35	0.80 (0.19–3.44)	0.769	0.98 (0.49–1.96)	0.948	1.00 [Ref]	1.25 (0.78–2.01)	0.350	1.30 (0.82–2.05)	0.264	1.56 (1.01–2.41)	0.047
Previous preterm births group											
No	1.05 (0.63–1.73)	0.862	1.02 (0.75–1.39)	0.911	1.00 [Ref]	1.04 (0.82–1.31)	0.768	1.19 (0.95–1.49)	0.130	1.21 (0.97–1.51)	0.088
Yes	0.41 (0.12–1.43)	0.164	1.27 (0.67–2.41)	0.466	1.00 [Ref]	0.97 (0.55–1.70)	0.904	0.79 (0.45–1.39)	0.409	0.87 (0.50–1.52)	0.633

*OR* odds ratio, *CI* confidence interval, *Ref* reference, *ICU* intensive care unit; OR shows the results of multivariate logistic regression analysis after adjusting for age (unadjusted in age group analysis), race, education level, marital status, weight gain, smoking before pregnancy, smoking during pregnancy, prenatal care, pre-pregnancy BMI, pre-pregnancy diabetes, gestational diabetes, pre-pregnancy hypertension, gestation hypertension, eclampsia, assisted reproductive treatment, gestational age, clinical chorioamnionitis or maternal fever during labor, and previous preterm birth (unadjusted in previous preterm births group analysis)

In the analysis of the effect of IPI on specific maternal adverse events, IPI of 36–59 months (OR = 0.88, 95%CI: 0.78–0.99) and ≥ 60 months (OR = 0.86, 95%CI: 0.76–0.97) were associated with a decreased risk of maternal transfusion. IPI of ≥ 60 months was related to a decreased risk of the ruptured uterus in women aged < 35 years (OR = 0.59, 95%CI: 0.45–0.78) and without previous preterm birth (OR = 0.65, 95%CI: 0.50–0.86). No association was observed between IPI and the risk of unplanned hysterectomy (all *P* > 0.05). IPI of ≥ 60 months (OR = 1.56, 95%CI: 1.01–2.41) was found to be associated with an increased risk of ICU admission in women aged ≥ 35 years.

### Association between IPI and neonatal adverse events

Table 5 demonstrates the impact of IPI on neonatal adverse events. After adjusting for confounders, IPI of ≤ 11 months (OR = 1.14, 95%CI: 1.07–1.21), 12–17 months (OR = 1.07, 95%CI: 1.03–1.10), and ≥ 60 months (OR = 1.05, 95%CI: 1.02–1.08) were related to an increased risk of neonatal adverse events compared with IPI of 18–23 months. Stratified analyses based on age and previous preterm births indicated that IPI of ≤ 11 months and 12–17 months were observed to be associated with an increased risk of neonatal adverse events in women aged < 35 years and women without previous preterm birth, and IPI of ≥ 60 months was found to be related to an increased risk of neonatal adverse events in women aged ≥ 35 years and women without previous preterm birth (all* P* < 0.05).

**Table 5:** Effect of interpregnancy interval (IPI) on neonatal adverse events

Outcome	Interpregnancy interval										
	≤ 11 month		12 ~ 17 month		18 ~ 23 month	24 ~ 35 month		36 ~ 59 month		≥ 60 month	
	OR (95% CI)	*P*	OR (95% CI)	*P*	OR (95% CI)	OR (95% CI)	*P*	OR (95% CI)	*P*	OR (95% CI)	*P*
**Neonatal adverse events**											
Total population	1.14 (1.07–1.21)	< 0.001	1.07 (1.03–1.10)	< 0.001	1.00 [Ref]	0.99 (0.97–1.02)	0.714	0.99 (0.97–1.02)	0.732	1.05 (1.02–1.08)	< 0.001
Age group											
< 35	1.14 (1.07–1.21)	< 0.001	1.06 (1.02–1.11)	0.002	1.00 [Ref]	0.99 (0.97–1.03)	0.761	0.99 (0.96–1.01)	0.321	1.03 (1.01–1.06)	0.089
≥ 35	1.15 (0.96–1.37)	0.130	1.06 (0.97–1.16)	0.164	1.00 [Ref]	0.99 (0.94–1.06)	0.941	1.04 (0.98–1.10)	0.222	1.10 (1.04–1.17)	< 0.001
Previous preterm births group											
No	1.14 (1.07–1.21)	< 0.001	1.06 (1.02–1.10)	0.002	1.00 [Ref]	0.99 (0.97–1.02)	0.733	0.99 (0.97–1.02)	0.683	1.05 (1.02–1.08)	< 0.001
Yes	1.17 (0.95–1.44)	0.135	1.13 (0.99–1.29)	0.071	1.00 [Ref]	0.99 (0.89–1.10)	0.891	1.01 (0.91–1.12)	0.856	1.03 (0.93–1.14)	0.592
**Low birth weight**											
Total population	1.38 (1.26–1.52)	< 0.001	1.07 (1.01–1.14)	0.035	1.00 [Ref]	1.07 (1.02–1.13)	0.006	1.22 (1.17–1.28)	< 0.001	1.51 (1.44–1.58)	< 0.001
Age group											
< 35	1.39 (1.26–1.53)	< 0.001	1.09 (1.02–1.16)	0.015	1.00 [Ref]	1.09 (1.03–1.15)	0.001	1.24 (1.18–1.31)	< 0.001	1.51 (1.44–1.59)	< 0.001
≥ 35	1.39 (1.05–1.83)	0.022	0.97 (0.83–1.13)	0.669	1.00 [Ref]	0.99 (0.89–1.11)	0.964	1.16 (1.04–1.29)	0.007	1.49 (1.35–1.65)	< 0.001
Previous preterm births group											
No	1.41 (1.28–1.56)	< 0.001	1.07 (1.01–1.14)	0.053	1.00 [Ref]	1.06 (1.01–1.12)	0.031	1.22 (1.16–1.28)	< 0.001	1.51 (1.44–1.58)	< 0.001
Yes	1.24 (0.98–1.56)	0.067	1.07 (0.91–1.26)	0.390	1.00 [Ref]	1.14 (1.01–1.30)	0.059	1.23 (1.08–1.40)	0.002	1.51 (1.33–1.71)	< 0.001
**Premature birth**											
Total population	0.99 (0.44–2.24)	0.996	1.01 (0.61–1.68)	0.963	1.00 [Ref]	1.01 (0.68–1.49)	0.974	0.98 (0.67–1.44)	0.930	1.01 (0.69–1.48)	0.960
Age group											
< 35	1.01 (0.43–2.38)	0.976	1.02 (0.59–1.76)	0.940	1.00 [Ref]	1.01 (0.65–1.56)	0.974	0.98 (0.64–1.49)	0.918	1.01 (0.66–1.52)	0.996
≥ 35	0.93 (0.08–10.44)	0.956	0.98 (0.27–3.50)	0.972	1.00 [Ref]	1.01 (0.41–2.44)	0.998	0.99 (0.42–2.36)	0.990	1.01 (0.44–2.31)	0.980
Previous preterm births group											
No	0.99 (0.42–2.35)	0.979	1.01 (0.59–1.73)	0.959	1.00 [Ref]	1.01 (0.66–1.52)	0.982	0.98 (0.66–1.47)	0.937	1.01 (0.68–1.51)	0.958
Yes	1.07 (0.12–9.69)	0.951	1.01 (0.23–4.44)	0.987	1.00 [Ref]	1.01 (0.31–3.36)	0.981	0.97 (0.30–3.15)	0.966	1.01 (0.31–3.26)	0.984
**Apgar score at 5 min < 7**											
Total population	1.10 (0.96–1.28)	0.177	1.03 (0.94–1.14)	0.476	1.00 [Ref]	1.01 (0.93–1.08)	0.860	1.01 (0.94–1.08)	0.884	1.03 (0.95–1.10)	0.497
Age group											
< 35	1.08 (0.92–1.26)	0.345	1.03 (0.93–1.13)	0.631	1.00 [Ref]	1.01 (0.92–1.09)	0.996	0.98 (0.91–1.06)	0.638	0.98 (0.90–1.06)	0.592
≥ 35	1.49 (0.98–2.27)	0.061	1.12 (0.87–1.43)	0.385	1.00 [Ref]	1.03 (0.86–1.24)	0.730	1.10 (0.92–1.31)	0.291	1.11 (0.94–1.31)	0.214
Previous preterm births group											
No	1.12 (0.96–1.31)	0.151	1.03 (0.93–1.14)	0.544	1.00 [Ref]	1.01 (0.93–1.09)	0.892	0.99 (0.92–1.07)	0.868	1.03 (0.96–1.11)	0.417
Yes	1.03 (0.71–1.51)	0.862	1.06 (0.80–1.41)	0.674	1.00 [Ref]	1.02 (0.81–1.30)	0.836	1.11 (0.88–1.39)	0.377	0.96 (0.77–1.21)	0.734
**Abnormal conditions of the newborn**											
Total population	1.18 (1.11–1.25)	< 0.001	1.07 (1.03–1.11)	< 0.001	1.00 [Ref]	1.02 (0.99–1.05)	0.278	1.02 (0.99–1.05)	0.145	1.05 (1.02–1.08)	< 0.001
Age group											
< 35	1.18 (1.10–1.26)	< 0.001	1.06 (1.02–1.11)	0.003	1.00 [Ref]	1.02 (0.99–1.06)	0.207	1.02 (0.99–1.06)	0.194	1.06 (1.02–1.09)	< 0.001
≥ 35	1.14 (0.95–1.38)	0.162	1.08 (0.98–1.19)	0.102	1.00 [Ref]	1.01 (0.95–1.08)	0.731	1.04 (0.98–1.11)	0.211	1.08 (1.02–1.15)	0.011
Previous preterm births group											
No	1.17 (1.10–1.25)	< 0.001	1.07 (1.03–1.11)	0.001	1.00 [Ref]	1.01 (0.98–1.05)	0.352	1.02 (0.99–1.05)	0.135	1.06 (1.03–1.09)	< 0.001
Yes	1.20 (0.99–1.45)	0.061	1.09 (0.96–1.23)	0.204	1.00 [Ref]	1.02 (0.92–1.13)	0.669	0.99 (0.89–1.10)	0.845	0.99 (0.90–1.10)	0.885

*OR* odds ratio; *CI* confidence interval, *Ref* reference; OR shows the results of multivariate logistic regression analysis after adjusting for age (unadjusted in age group analysis), race, weight gain, smoking before pregnancy, smoking during pregnancy, prenatal care, pre-pregnancy BMI, pre-pregnancy diabetes, gestational diabetes, pre-pregnancy hypertension, gestation hypertension, eclampsia, assisted reproductive treatment, gestational age, clinical chorioamnionitis or maternal fever during labor, and previous preterm birth (unadjusted in previous preterm births group analysis)

In the analysis of the effect of IPI on specific neonatal adverse events, IPI of ≤ 11 months (OR = 1.38, 95%CI: 1.26–1.52), 12–17 months (OR = 1.07, 95%CI: 1.01–1.14), 24–35 months (OR = 1.07, 95%CI: 1.02–1.13), 36–59 months (OR = 1.22, 95%CI: 1.17–1.28), and ≥ 60 months (OR = 1.51, 95%CI: 1.44–1.58) were related to an increased risk of low birth weight. No associations of IPI with the risk of premature birth and Apgar score at 5 min < 7 were found (all *P* > 0.05). IPI of ≤ 11 months (OR = 1.18, 95%CI: 1.11–1.25), 12–17 months (OR = 1.07, 95%CI: 1.03–1.11), and ≥ 60 months (OR = 1.05, 95%CI: 1.02–1.08) were found to be associated with an increased risk of abnormal conditions of the neonatal.

## Discussion

This study analyzed the effect of the IPI after maternal cesarean delivery on the risk of repeat cesarean delivery, maternal and neonatal adverse events. Both short and long IPIs were associated with an increased risk of repeat cesarean delivery. Long IPI was found to be related to a decreased risk of maternal adverse events in women aged < 35 years. In the association between IPI and neonatal adverse events, short and long IPIs were related to an increased risk of neonatal adverse events, especially among women aged < 35 years.

The delivery model of the first pregnancy is associated with subsequent maternal pregnancy outcomes. Winsen et al. demonstrated that emergency cesarean delivery in the first pregnancy was related to a higher rate of preterm birth and an increased risk of admission to a neonatal unit in subsequent pregnancies compared to vaginal delivery [13]. Previous studies have explored the impact of IPI on subsequent pregnancy outcomes [5, 9, 12, 23]. Both short and long IPI were reported to be associated with increased risk of preterm birth, low, birth weight, small-for-gestational-age birth, and neonatal ICU admission [5, 9, 24, 25]. However, previous studies have not distinguished the effect of IPI after different delivery models on subsequent pregnancy outcomes. This study analyzed the effect of maternal IPI after cesarean delivery on maternal and neonatal outcomes in subsequent pregnancies. Our results demonstrated that both short and long IPIs were associated with an increased risk of repeat cesarean delivery. This association persisted among women who experienced TOLAC. This result may be related to the fact that women who had a previous cesarean delivery were more likely to have a subsequent cesarean delivery [17]. This may also be related to the worldwide increase in the prevalence of cesarean delivery [26]. Our results showed that 88.91% of women who had a first cesarean delivery underwent a repeat cesarean delivery in subsequent pregnancies. The increase in cesarean rates is influenced by many factors including demand for cesarean delivery, advanced maternal age at first pregnancy, and a decrease in the number of patients attempting vaginal delivery after cesarean delivery [27–29]. One possible reason for the high rate of repeat cesarean delivery in women with long IPI is maternal age. Long IPI increases maternal age, and women of advanced maternal age have a higher rate of cesarean delivery than non-advanced maternal age women [29, 30]. In addition, the effect of a long IPI on the risk of repeat cesarean delivery may be related to time-limited physiological adaptations of the reproductive system resulting from pregnancy (e.g., increased blood flow to the uterus) [8, 31]. As the interval between pregnancies increases, these adaptations may regress and maternal physiological characteristics may revert to those of primiparous women, which may cause an increase in the odds of cesarean delivery [8].

The relationship between IPI and maternal adverse events showed that a long IPI (≥ 60 months) was found to be associated with a decreased risk of maternal transfusion and ruptured uterus in women aged < 35 years. Previous studies demonstrated that women who gave birth for the first time at age 30 years or older had a shorter IPI compared to women who started childbearing at younger ages [32, 33]. However, our results were not consistent with those of previous studies [4, 22]. Garg et al. found that women with an IPI < 6 months were related to an increased risk of maternal transfusion compared to women with an IPI of 18–23 months, while no statistical significance was observed in women with an IPI ≥ 60 months [22]. Silva et al. indicated that the risk of maternal transfusion followed a U-shaped curve with increasing IPI compared to IPI at 18–23 months [4]. Possible explanation for the inconsistency of our results was the difference in the study population and the adjusted confounders. Our study focused on women whose first pregnancy was by cesarean delivery and more comprehensively considered the effects of confounders such as pre-pregnancy BMI, weight gain, smoking status, and previous medical history. In addition, previous studies have not conducted stratified analysis according to age. In the association between IPI and adverse neonatal events, both short and long IPIs were related to an increased risk of neonatal adverse events, which was consistent with previous studies [5, 9, 24, 25]. Mignini et al. showed that longer intervals of > 72 months was associated with pre-eclampsia, fetal death, and low birth weight [7]. Our results suggested that the effect of IPI on adverse neonatal events was more frequent in women aged < 35 years. Schummers et al. found that the risk of adverse fetal and infant outcomes was more pronounced in women aged 20 to 34 years than in women aged 35 years or older [12].

Our study explored the effect of IPI on maternal and neonatal outcomes in subsequent pregnancies in women whose first delivery was by cesarean delivery using large-sample multicenter data from the NVSS database. However, our study also has several limitations. First, the study population of subjects whose first delivery was by cesarean delivery reduced the sample size, which may have resulted in reduced statistical power to detect the association between IPI and maternal and neonatal outcomes. Second, this study only included pregnant women with 2 consecutive singleton pregnancies and a first delivery by cesarean delivery, and the results may not be generalizable to women with multiple deliveries or multiple cesarean deliveries. Third, pregnancy outcomes and possible influencing factors recorded in the medical records were included in this study as far as possible, but there were still some confounders such as cervical maturity, duration of labor, and information on vitamins/iron/folic supplements were not recorded. Fourth, we did not consider the bias from the long study duration such as changes in obstetrical care/protocols during the study period. Fifth, although we considered clinical chorioamnionitis or maternal fever during labor as a confounder, other information on the risk of invasive placental diagnosis associated with IPI was not considered due to database limitations. Sixth, the mode of cesarean delivery, the number of layers for uterine closure, and the TOLAC protocols were not available due to the absence of relevant records in the database.

## Conclusions

The associations between IPI and maternal and neonatal outcomes in subsequent pregnancies was investigated in women whose first delivery was by cesarean delivery. Both short and long IPI were associated with an increased risk of repeat cesarean delivery and neonatal adverse events. Among women aged < 35 years, an IPI more than 36 months after the first cesarean delivery was associated with a low risk of maternal transfusion and ruptured uterus.

## Supplementary Information

**Additional file 1: Supplement Table 1.** Univariate logistic regression analysis between covariates and the risk of cesarean delivery. **Supplement Table 2.** Univariate logistic regression analysis between covariates and the risk of any maternal adverse events. **Supplement Table 3.** Univariate logistic regression analysis between covariates and the risk of any neonatal adverse events.

## Data Availability

The datasets generated and/or analyzed during the current study are available in the NVSS database, https://www.cdc.gov/nchs/nvss/index.htm.

## Abbreviations

IPI: Interpregnancy interval
NICU: Neonatal intensive care unit
NVSS: National Vital Statistics System
NCHS: National Center for Health Statistics
BMI: Body mass index
ICU: Intensive care unit
SD: Standard deviation
OR: Odds ratio
CI: Confidence interval
