# Supplementary material for: Association between interpregnancy interval and maternal and neonatal adverse outcomes in women with a cesarean delivery: a population-based study
Source: BMC Pregnancy Childbirth. 2023 Apr 25;23:284. doi: 10.1186/s12884-023-05600-x (PMC10127338; doi:10.1186/s12884-023-05600-x)
Supplement: Supplementary file 1 — Additional file 1: Supplement Table 1. Univariate logistic regression analysis between covariates and the risk of cesarean delivery. Supplement Table 2. Univariate logistic regression analysis between covariates and the risk of any maternal adverse events. Supplement Table 3. Univariate logistic regression analysis between covariates and the risk of any neonatal adverse events. [file 12884_2023_5600_MOESM1_ESM.docx]

**Supplement Table 1**. Univariate logistic regression analysis between covariates and the risk of cesarean delivery.

| **Variables** | **β** | **S. E** | **OR (95% CI)** | ***P*** |
| --- | --- | --- | --- | --- |
| Age | 0.03 | 0.00 | 1.03 (1.03-1.03) | <0.001 |
| Race |  |  |  |  |
| White |  |  | Ref |  |
| Black | 0.02 | 0.01 | 1.02 (1.01-1.04) | 0.090 |
| Other | -0.11 | 0.02 | 0.90 (0.86-0.94) | <0.001 |
| Asian | -0.13 | 0.01 | 0.88 (0.85-0.90) | <0.001 |
| Education level |  |  |  |  |
| Less than high school |  |  | Ref |  |
| High school | 0.48 | 0.02 | 1.61 (1.53-1.68) | <0.001 |
| More than high school | 0.34 | 0.02 | 1.40 (1.34-1.47) | <0.001 |
| Missing | 0.32 | 0.04 | 1.37 (1.27-1.48) | <0.001 |
| Marital status |  |  |  |  |
| Married |  |  | Ref |  |
| Unmarried | 0.19 | 0.01 | 1.20 (1.18-1.22) | <0.001 |
| Missing | 0.20 | 0.01 | 1.22 (1.19-1.25) | <0.001 |
| Weight gain | 0.00 | 0.00 | 1.01 (1.01-1.01) | <0.001 |
| Smoking before pregnancy |  |  |  |  |
| No |  |  | Ref |  |
| Yes | 0.47 | 0.02 | 1.60 (1.55-1.65) | <0.001 |
| Smoking during pregnancy |  |  |  |  |
| No |  |  | Ref |  |
| Yes | 0.47 | 0.02 | 1.61 (1.55-1.67) | <0.001 |
| Prenatal care |  |  |  |  |
| No |  |  | Ref |  |
| Yes | 0.37 | 0.04 | 1.44 (1.35-1.54) | <0.001 |
| Pre-pregnancy BMI | 0.05 | 0.00 | 1.05 (1.05-1.06) | <0.001 |
| Pre-pregnancy diabetes |  |  |  |  |
| No |  |  | Ref |  |
| Yes | 0.90 | 0.04 | 2.47 (2.28-2.68) | <0.001 |
| Gestational diabetes |  |  |  |  |
| No |  |  | Ref |  |
| Yes | 0.35 | 0.02 | 1.42 (1.38-1.46) | <0.001 |
| Pre-pregnancy hypertension |  |  |  |  |
| No |  |  | Ref |  |
| Yes | 0.82 | 0.03 | 2.27 (2.14-2.41) | <0.001 |
| Gestational hypertension |  |  |  |  |
| No |  |  | Ref |  |
| Yes | 0.48 | 0.02 | 1.61 (1.56-1.67) | <0.001 |
| Eclampsia |  |  |  |  |
| No |  |  | Ref |  |
| Yes | 0.48 | 0.09 | 1.61 (1.35-1.91) | <0.001 |
| Assisted reproductive treatment |  |  |  |  |
| No |  |  | Ref |  |
| Yes | 0.54 | 0.04 | 1.71 (1.58-1.86) | <0.001 |
| Gestational age | -0.08 | 0.00 | 0.92 (0.92-0.93) | <0.001 |
| Clinical chorioamnionitis or maternal fever during labor |  |  |  |  |
| No |  |  | Ref |  |
| Yes | -1.97 | 0.03 | 0.14 (0.13-0.15) | <0.001 |
| Previous preterm birth |  |  |  |  |
| No |  |  | Ref |  |
| Yes | -0.23 | 0.02 | 0.79 (0.77-0.82) | <0.001 |

Note: OR, odds ratio; CI, confidence interval; Ref, reference; S. E, standard error.

**Supplement Table 2**. Univariate logistic regression analysis between covariates and the risk of any maternal adverse events.

| **Variables** | **β** | **S. E** | **OR (95% CI)** | ***P*** |
| --- | --- | --- | --- | --- |
| Age | -0.01 | 0.00 | 0.99 (0.99-0.99) | 0.054 |
| Race |  |  |  |  |
| White |  |  | Ref |  |
| Black | 0.43 | 0.04 | 1.53 (1.43-1.64) | <0.001 |
| Other | 0.31 | 0.07 | 1.37 (1.19-1.57) | <0.001 |
| Asian | 0.13 | 0.05 | 1.14 (1.04-1.25) | 0.006 |
| Education level |  |  |  |  |
| Less than high school |  |  | Ref |  |
| High school | -0.15 | 0.09 | 0.86 (0.72-1.03) | 0.110 |
| More than high school | -0.34 | 0.09 | 0.71 (0.60-0.85) | <0.001 |
| Missing | -0.07 | 0.15 | 0.93 (0.70-1.25) | 0.631 |
| Marital status |  |  |  |  |
| Married |  |  | Ref |  |
| Unmarried | 0.19 | 0.03 | 1.21 (1.15-1.29) | <0.001 |
| Missing | -0.45 | 0.05 | 0.64 (0.58-0.71) | <0.001 |
| Weight gain | -0.01 | 0.00 | 0.99 (0.99-0.99) | <0.001 |
| Smoking before pregnancy |  |  |  |  |
| No |  |  | Ref |  |
| Yes | 0.21 | 0.05 | 1.23 (1.12-1.36) | <0.001 |
| Smoking during pregnancy |  |  |  |  |
| No |  |  | Ref |  |
| Yes | 0.21 | 0.06 | 1.23 (1.11-1.37) | <0.001 |
| Prenatal care |  |  |  |  |
| No |  |  | Ref |  |
| Yes | -0.92 | 0.10 | 0.40 (0.33-0.48) | <0.001 |
| Pre-pregnancy BMI | -0.01 | 0.00 | 0.99 (0.99-0.99) | <0.001 |
| Pre-pregnancy diabetes |  |  |  |  |
| No |  |  | Ref |  |
| Yes | 0.85 | 0.07 | 2.35 (2.03-2.72) | <0.001 |
| Gestational diabetes |  |  |  |  |
| No |  |  | Ref |  |
| Yes | 0.13 | 0.05 | 1.14 (1.04-1.25) | 0.006 |
| Pre-pregnancy hypertension |  |  |  |  |
| No |  |  | Ref |  |
| Yes | 0.70 | 0.06 | 2.00 (1.78-2.26) | <0.001 |
| Gestational hypertension |  |  |  |  |
| No |  |  | Ref |  |
| Yes | 0.58 | 0.05 | 1.79 (1.64-1.96) | <0.001 |
| Eclampsia |  |  |  |  |
| No |  |  | Ref |  |
| Yes | 1.30 | 0.15 | 3.68 (2.74-4.93) | <0.001 |
| Assisted reproductive treatment |  |  |  |  |
| No |  |  | Ref |  |
| Yes | 0.91 | 0.08 | 2.49 (2.12-2.94) | <0.001 |
| Gestational age | -0.17 | 0.01 | 0.84 (0.84-0.85) | <0.001 |
| Clinical chorioamnionitis or maternal fever during labor |  |  |  |  |
| No |  |  | Ref |  |
| Yes | 1.43 | 0.08 | 4.17 (3.55-4.90) | <0.001 |
| Previous preterm birth |  |  |  |  |
| No |  |  | Ref |  |
| Yes | 0.77 | 0.05 | 2.16 (1.97-2.37) | <0.001 |

Note: OR, odds ratio; CI, confidence interval; Ref, reference; S. E, standard error.

**Supplement Table 3**. Univariate logistic regression analysis between covariates and the risk of any neonatal adverse events.

| **Variables** | **β** | **S. E** | **OR (95% CI)** | ***P*** |
| --- | --- | --- | --- | --- |
| Age | 0.00 | 0.00 | 0.99 (0.99-0.99) | <0.001 |
| Race |  |  |  |  |
| White |  |  | Ref |  |
| Black | 0.44 | 0.01 | 1.55 (1.53-1.57) | <0.001 |
| Other | 0.19 | 0.02 | 1.21 (1.18-1.25) | <0.001 |
| Asian | -0.13 | 0.01 | 0.88 (0.86-0.90) | <0.001 |
| Education level |  |  |  |  |
| Less than high school |  |  | Ref |  |
| High school | 0.03 | 0.02 | 1.03 (0.99-1.07) | 0.137 |
| More than high school | -0.23 | 0.02 | 0.79 (0.76-0.82) | <0.001 |
| Missing | -0.19 | 0.04 | 0.83 (0.78-0.89) | <0.001 |
| Marital status |  |  |  |  |
| Married |  |  | Ref |  |
| Unmarried | 0.34 | 0.01 | 1.41 (1.39-1.43) | <0.001 |
| Missing | -0.23 | 0.01 | 0.80 (0.78-0.81) | <0.001 |
| Weight gain | -0.01 | 0.00 | 0.99 (0.99-0.99) | <0.001 |
| Smoking before pregnancy |  |  |  |  |
| No |  |  | Ref |  |
| Yes | 0.40 | 0.01 | 1.50 (1.47-1.53) | <0.001 |
| Smoking during pregnancy |  |  |  |  |
| No |  |  | Ref |  |
| Yes | 0.49 | 0.01 | 1.63 (1.60-1.67) | <0.001 |
| Prenatal care |  |  |  |  |
| No |  |  | Ref |  |
| Yes | -0.98 | 0.03 | 0.38 (0.36-0.40) | <0.001 |
| Pre-pregnancy BMI | 0.02 | 0.00 | 1.01 (1.01-1.02) | <0.001 |
| Pre-pregnancy diabetes |  |  |  |  |
| No |  |  | Ref |  |
| Yes | 1.34 | 0.02 | 3.81 (3.68-3.95) | <0.001 |
| Gestational diabetes |  |  |  |  |
| No |  |  | Ref |  |
| Yes | 0.39 | 0.01 | 1.47 (1.44-1.50) | <0.001 |
| Pre-pregnancy hypertension |  |  |  |  |
| No |  |  | Ref |  |
| Yes | 1.04 | 0.01 | 2.83 (2.75-2.91) | <0.001 |
| Gestational hypertension |  |  |  |  |
| No |  |  | Ref |  |
| Yes | 1.09 | 0.01 | 2.97 (2.92-3.03) | <0.001 |
| Eclampsia |  |  |  |  |
| No |  |  | Ref |  |
| Yes | 1.47 | 0.05 | 4.35 (3.98-4.76) | <0.001 |
| Assisted reproductive treatment |  |  |  |  |
| No |  |  | Ref |  |
| Yes | 0.31 | 0.03 | 1.36 (1.30-1.43) | <0.001 |
| Gestational age | -0.98 | 0.00 | 0.38 (0.37-0.38) | <0.001 |
| Clinical chorioamnionitis or maternal fever during labor |  |  |  |  |
| No |  |  | Ref |  |
| Yes | 1.30 | 0.03 | 3.67 (3.49-3.87) | <0.001 |
| Previous preterm birth |  |  |  |  |
| No |  |  | Ref |  |
| Yes | 1.17 | 0.01 | 3.23 (3.16-3.30) | <0.001 |

Note: OR, odds ratio; CI, confidence interval; Ref, reference; S. E, standard error.
